# Supplementary material for: Mental health and quality of life among healthcare professionals during the COVID‐19 pandemic in India
Source: Brain Behav. 2020 Sep 11;10(11):e01837. doi: 10.1002/brb3.1837 (PMC7667343; doi:10.1002/brb3.1837)
Supplement: Supplementary file 1 — Table S1 [file BRB3-10-e01837-s001.docx]

| Supplementary Table 1. Prevalence of mental health symptoms by socio-demographic characteristics. | | | | | | | |
| --- | --- | --- | --- | --- | --- | --- | --- |
| **Characteristics** | **N** | **Any Depressive**  **Symptoms**  n (%) | **Any Anxiety**  **Symptoms**  n (%) | **Moderate to Severe Depression**  % (95% CI) | **Moderate to Severe Anxiety**  % (95% CI) | **Moderate to Severe Depression and Anxiety Combined**  % (95% CI) | **Low QoL**  % (95% CI) |
| Overall n  Overall % | 197  (100%) | 92  (47%) | 98  (50%) | 44  22% (17% - 29%) | 56  29% (23% - 36%) | 33  17% (12% - 23%) | 89  45% (38% - 52%) |
| Gender  Male  Female | 101 (51%)  96 (49%) | 48 (48%)  44 (47%) | 54 (55%)  44 (46%) | 22% (14% - 31%)  23% (15% - 32%) | 25% (17% - 35%)  32% (23% - 42%) | 17% (10% - 26%)  17% (10% - 26%) | 49% (39% - 59%)  42% (32% - 52%) |
| Age (years)  ≤ 30  31 – 40  > 40 | 81 (41%)  73 (37%)  43 (22%) | 42 (52%)  32 (44%)  18 (42%) | 38 (48%)  39 (54%)  21 (50%) | 30% (20% - 41%)  19% (11% - 30%)  14% (5% - 28%) | 33% (22% - 44%)  28% (18% - 40%)  24% (12% - 39%) | 22% (14% - 33%)  14% (7% - 24%)  12% (4% - 25%) | 42% (31% - 53%)  45% (34% - 57%)  51% (35% - 67%) |
| Marital Status  Married  Single^a^ | 119 (60%)  78 (40%) | 49 (41%)  43 (55%) | 57 (49%)  41 (53%) | 16% (10% - 24%)  32% (22% - 44%) | 26% (18% - 35%)  34% (23% - 45%) | 12% (6% - 19%)  24% (15% - 35%) | 46% (37% - 56%)  44% (32% - 55%) |
| Direct COVID-19 Care  No  Yes | 26 (13%)  171 (87%) | 13 (50%)  79 (46%) | 9 (36%)  89 (53%) | 12% (2% - 30%)  24% (18% - 31%) | 24% (9% - 45%)  30% (23% - 37%) | 12% (2% - 30%)  18% (12% - 24%) | 50% (30% - 70%)  44% (37% - 52%) |
| Avg. Number of COVID-19 Patients/Day  < 10  > 10 | 113 (57%)  84 (43%) | 51 (45%)  41 (49%) | 51 (46%)  47 (56%) | 21% (14% - 30%)  24% (15% - 34%) | 25% (18% - 35%)  33% (23% - 44%) | 15% (9% - 23%)  19% (11% - 29%) | 42% (33% - 52%)  49% (38% 60%) |
| City  Pune  Out of Pune | 97 (49%)  100 (51%) | 44 (45%)  48 (48%) | 49 (50%)  49 (51%) | 25% (17% - 35%)  20% (12% - 29%) | 31% (22% – 41%)  27% (19% - 37%) | 19% (12% - 28%)  14% (8% - 23%) | 44% (34% - 54%)  46% (36% - 57%) |
| Hospital Setting  Private  Public | 67 (34%)  130 (66%) | 32 (48%)  60 (46%) | 30 (45%)  68 (53%) | 18% (10% - 29%)  25% (17% - 33%) | 23% (13% - 35%)  32% (24% - 41%) | 12% (5% - 22%)  19% (13% - 27%) | 46% (34% - 59%)  45% (36% - 54%) |
| HCP Role  Paraclinical^b^  Resident/Intern  Nurse  Physician | 66 (34%)  26 (13%)  58 (29%)  47 (24%) | 24 (36%)  10 (38%)  29 (50%)  29 (62%) | 31 (47%)  12 (48%)  28 (49%)  27 (59%) | 15% (4% - 35%)  26% (15% - 39%)  28% (16% - 43%)  18% (10% - 30%) | 24% (9% - 45%)  33% (21% - 47%)  30% (18% - 46%)  26% (16% - 38%) | 12% (2% - 30%)  22% (13% - 35%)  15% (6% - 28%)  15% (8% - 26%) | 58% (37% - 77%)  41% (29% - 55%)  45% (30% - 60%)  44% (32% - 57%) |
| Years of Experience  < 5  5 – 10  > 10 | 92 (47%)  43 (22%)  62 (31%) | 23 (53%)  43 (47%)  26 (42%) | 25 (58%)  41 (46%)  32 (52%) | 27% (18% - 37%)  21% (10% - 36%)  16% (8% - 28%) | 30% (21% - 41%)  33% (19% - 49%)  25% (14% - 37%) | 21% (13% - 30%)  16% (7% - 31%)  11% (5% - 22%) | 42% (32% - 53%)  44% (29% - 60%)  50% (37% - 63%) |
| Abbreviation: HCP: Health Care professional  ^a^Single included HCPs who are unmarried, widowed, separated and divorced  ^b^Paraclinical HCPs included laboratory personnel, Radiologists, X-ray technicians and epidemiologists. | | | | | | | |
